# Simulated complexes formed from a set of postsynaptic proteins suggest a localised effect of a hypomorphic Shank mutation

**DOI:** 10.1186/s12868-024-00880-1

**Published:** 2024-07-06

**Authors:** Marcell Miski, Áron Weber, Krisztina Fekete-Molnár, Bence Márk Keömley-Horváth, Attila Csikász-Nagy, Zoltán Gáspári

**Affiliations:** 1 Faculty of Information Technology and Bionics, Pázmány Péter Catholic University, Budapest, Hungary; 2 Cytocast Hungary Kft, Budapest, Hungary

**Keywords:** Protein complex, Mutation, Systems biology, Protein interaction network, Protein:protein interaction, Gillespie algorithm, Postsynaptic density, Binding affinity

## Abstract

**Background:**

The postsynaptic density is an elaborate protein network beneath the postsynaptic membrane involved in the molecular processes underlying learning and memory. The postsynaptic density is built up from the same major proteins but its exact composition and organization differs between synapses. Mutations perturbing protein: protein interactions generally occurring in this network might lead to effects specific for cell types or processes, the understanding of which can be especially challenging.

**Results:**

In this work we use systems biology-based modeling of protein complex distributions in a simplified set of major postsynaptic proteins to investigate the effect of a hypomorphic Shank mutation perturbing a single well-defined interaction. We use data sets with widely variable abundances of the constituent proteins. Our results suggest that the effect of the mutation is heavily dependent on the overall availability of all the protein components of the whole network and no trivial correspondence between the expression level of the directly affected proteins and overall complex distribution can be observed.

**Conclusions:**

Our results stress the importance of context-dependent interpretation of mutations. Even the weakening of a generally occurring protein: protein interaction might have well-defined effects, and these can not easily be predicted based only on the abundance of the proteins directly affected. Our results provide insight on how cell-specific effects can be exerted by a mutation perturbing a generally occurring interaction even when the wider interaction network is largely similar.

**Supplementary Information:**

The online version contains supplementary material available at 10.1186/s12868-024-00880-1.

## Background

The synaptic theory states that the identity of the synapses in different cell types is a key component in establishing the complexity of brain functions, including learning and memory [1, 2] There are a number of experimental observations indicating that the postsynaptic density (PSD), utilizing the same major constituent proteins, can be highly variable depending on the abundance of its individual protein components [3]. The PSD is also capable of dynamic reorganization during the circadian cycle and upon stimuli [4], and its flexibility to do so has been proposed to play a key role in synaptic plasticity and network rewiring [5]. Despite our knowledge in a number of pairwise protein: protein interactions between postsynaptic proteins [6], the specific large-scale organization of the PSD is still largely elusive.

Mutations identified in proteins of the PSD in various neurological conditions might directly affect specific protein: protein interactions. Although the same proteins and interactions can occur in virtually all postsynaptic compartments, the effect of mutations is often specific to a brain region, to specific interaction partners or leads to the impairment or gain of defined functions instead of leading to the dysfunction of neural transmission in all or most cells [7]. This behavior is expected to be only interpretable by taking into account the complexity of the in vivo conditions.

The Shank protein family contains large modular scaffold proteins with both globular and long intrinsically disordered regions [8]. These proteins establish a number of diverse interactions with various postsynaptic proteins. Mutations affecting the availability and/or the structure of Shank proteins have been linked to many conditions [9] from autism spectrum disorder (ASD) [10] to Phelan-McDermid Syndrome (PMS) [11]. In these conditions, Shank3 haploinsufficiency [12], caused by either the complete loss of a copy of the gene or by the presence of a function-affecting mutation, is the most prevalent cause, although mutations in the Shank1 and Shank2 genes can also cause similar, although typically milder, phenotypes.

Besides state-of-the-art microscopic techniques [13, 14], simulation approaches might contribute to our more detailed understanding of the supramolecular structure of the PSD and its changes upon stimuli and mutations. We have previously described extensive simulations on protein complex formation using a simplified model of the PSD containing 7 major proteins. These simulations revealed that the correspondence between protein component abundance and the distribution of the complexes formed is nontrivial [15]. Specifically, the classification of the simulated PSDs based on the abundance of constituent proteins can largely differ from the classification based on the resulting protein complex distributions. Importantly, protein complexes represent a feature supposedly more closely linked to the biological function of the network than simple protein abundance. Our data set included instances of the same or similar brain regions with different expression levels of the individual proteins, and as such our previous work also models the effect of abundance-affecting mutations.

In this work, we address the question of whether hypomorphic mutations, i.e. those reducing but not completely abolishing a function, specifically a binding interaction, can cause measurable effects in our PSD model system. We have chosen the Shank1 PDZ domain as a model system because of its well-studied nature [16]. Mutations in this domain have been linked to ASD such as R736Q [10] and also have been identified in various cancers [17]. As a model hypomorphic mutation, we have chosen one that can be estimated to cause the decrease of a specific binding interaction between 2- and 10-fold. We use the same model system as in our previous work, composed of seven major PSD proteins, except for replacing Shank3 with Shank1 (NMDAR, AMPAR, PSD−95, SynGAP, GKAP, Homer1, Shank1) [15]. As previously, we performed our simulations on more than 500 brain regions with different protein levels [18].

For all simulations, the same set of interactions is defined, except for the one affected by the mutation, giving rise to the „wild type” and „mutated” scenarios. For each region, the input is the abundance of each protein and the ouput is the number of the different protein complexes formed. Our results suggest that weakening a single well-defined interaction does not affect the overall distribution of complexes in most investigated brain regions. However, in a small number of cases, the most informative protein complexes - defined based on their contribution to the overall diversity of the PSD complexes - show a significant change in their abundance. These results indicate that even when the same set of proteins is involved, the biological effect of a mutation can be highly specific depending on the cellular context.

## Methods

### Overview of the simulations

The aim of our simulations was to estimate the distributions of protein complexes formed by a set of proteins with different abundances (copy numbers). The set of proteins was a highly simplified PSD model including 7 major proteins, two receptors (AMPAR, NMDAR) and five scaffold proteins (Homer1, GKAP, SynGAP, and Shank1) with well-defined interactions between them (Tables S1, S2, S3).

For the simulation of protein complex formation, we have used an agent-based simulation tool (Cytocast), also applied in our previous study [15]. The underlying principle is the Gillespie algorithm, a related implementation of which was used to predict COVID-19 outcomes [19]. A previous implementation (SiComPre) was shown to effectively model protein complex distribution in whole cells [20].

### Data sets, setup and reproducibility

Overall, we have simulated protein complexes in 524 different data sets originating from 27 brain region types, as in our previous work [15] (Table S4). The source data sets contain mRNA expression levels [18] that we have translated into protein abundance by linear scaling of the values to the copy number of PSD-95 pporteins as described previously [15] (Table S5). We note that mRNMA levels can be used to approximate cellular protein abundance [21, 22]. Still, ours is a highly simplified approach that does not take any other factors like protein degradation into account, but is sufficient for our modeling purposes to obtain data sets with sufficient variation. The average copy numbers of the individual proteins, serving as input for the simulations, show a high variance (Table 1). It should be noted that no information about the individuals from which the data are derived is available, whether they can be considered as in a healthy or diseased condition. The raw input abundances can be found in the Supplementary material (Table S5). Dissociation constants were taken from the data described in the literature [23]. In our simulations, the dissociation constant was implemented by adjusting the unbinding rate as a result of a mutation, while not changing the binding rates. To estimate the reproducibility of the simulations, 40 simulations were performed for each data set. This number was deemed sufficient in terms of reproducibility, as with 40 repetitions we have reached a variance of 1 that did not improve upon more repetitions. In addition, Shapiro-Wilk test for normality, performed for the abundance of the most informative complex (see below) with Benjamini-Hochberg correction [24] indicated that the normal distribution of complex abundances can be assumed (can not be rejected) in all 524 regions when using 40 simulations.

**Table 1:** Statistics of input protein abundance derived from mRNA expression levels from reference [18]

Protein	Minimum	Maximum	Average	Deviation
NMDAR	0	95	16.84	17.87
AMPAR	1	688	126.81	78.39
PSD-95	36	1067	328.09	159.21
SynGAP	11	359	1102.21	60.13
GKAP	2	367	82.62	65.50
Shank1	1	388	69.20	59.40
Homer1	1	124	21.98	17.20

To make testing our calculations and setups possible, we have set up a web interface, available at https://psdcomplexsim.cytocast.com.

### Choice and modeling of the Shank1 R743H mutation

In order to use a well-studied interaction, we have chosen the Shank1 PDZ domain and its interaction with the C-terminus of GKAP. The Shank1 PDZ is a globular domain that has been characterized extensively [25]. Moreover, several mutations in the Shank1 PDZ domain has been observed in patients with Autism Spectrum Disorder (ASD) [10]. There are also mutations for this domain listed in the COSMIC database [17], and as these are available in an organized format, we decided to use this source to explore several mutations.

Our goal was to model a moderate yet measurable effect of a hypomorphic mutation that weakens but does not abolish binding. It is not trivial to estimate the perturbation effect of a mutation, especially for one outside the primary ligand binding site, Therefore, we have used the change in domain stability as predicted by the NeEMO method [26] and have used this change to estimate the weakening of the interaction by assuming less stable apo and holo structures.

**Fig. 1:**
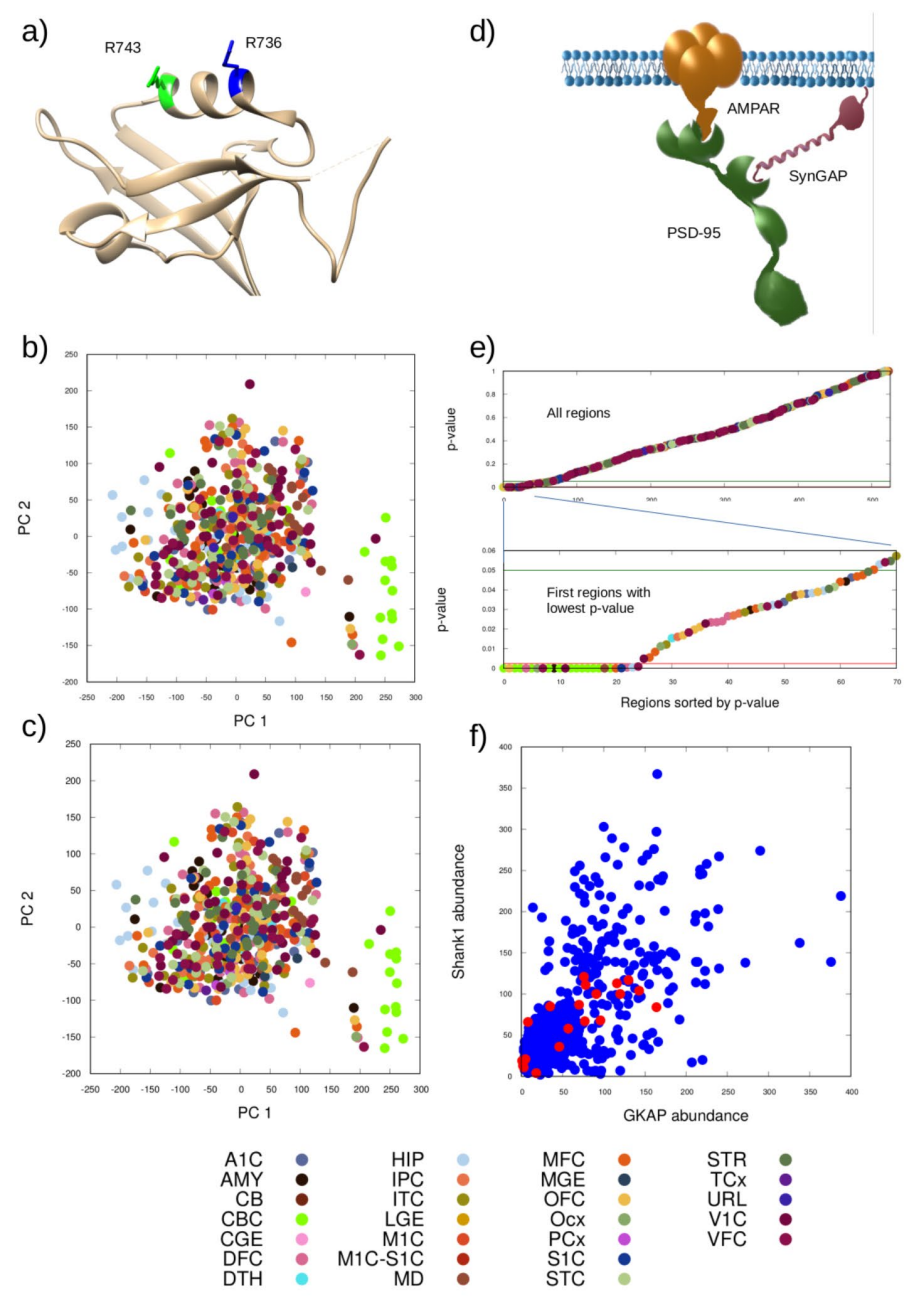
(**a**) Position of the mutation selected (R743H, green) and a similar one reported in ASD (R736Q, blue) on the ribbon representation of the Shank1 PZ domain (PDB ID 6YWZ). Both arginines are located on the α2 helix flanking the ligand binding groove. Principal component analysis of the obtained protein complex distributions for (**b**) the wild-type and (**c**) the mutant scenarios investigated. Different colors denote different brain regions according to the key at the bottom. (**d**) Schematic depiction of the most informative complex according to the PCA (AMPAR/PSD-95/SynGAP). (**e**) *P*-values describing the change upon the mutation relative to the wild-type, the value for the most informative complex is shown in increasing order from left to right, colored by the region type (key at the bottom) The green line denotes the 0.05 significance limit while the red line the limit of 0.0024 obtained using the Benjamini-Hochberg correction. (**f**) Abundance of Shank1 and GKAP, the two proteins in the interaction affected by the mutation, in the input data sets. Red circles indicate data sets where the abundance of the most informative complex changed significantly in the output using the Benjamini-Hochberg correction

For our modeling purposes, we have chosen the R743H mutation for which we have estimated a 5.5-fold decrease in the binding affinity, modeled as a 5.5-fold increase for the dissociation rate of the Shank1 PDZ: GKAP interaction in our setup. This value is regarded as a good compromise between a minimally observable 2-fold change and changing the value by one order of magnitude, a more pronounced change. The arginine affected by this mutation is located on the C-terminal end of helix 2, whereas the R736Q mutation, described in ASD, alters an arginine at the N-terminus of the same helix (Fig. 1a). Both arginines point away from the immediate ligand binding site, thus, both mutations are expected to perturb the binding interaction indirectly.

### Analysis of the simulation results

In our system composed of multivalent proteins, the identity of the protein complexes formed is not only determined by their composition but also the exact topology, i.e. how the constituent proteins interact with each other, rather also by the binding sites actually participate in the interactions. Complexes with the same protein composition but different binding patterns should therefore be distinguished during the analysis of the simulation results.

All protein complexes observed were identified and enumerated, and each was assigned a unique identifier. The simulated results for each input data set can be represented by a point in a multidimensional space where each coordinate represents the abundance of a given protein complex. Thus, a protein complex distribution can be described as the linear combination of the emerging protein complexes (Eq. 1):

1 $$c\in {R}^{n},c=\sum\limits_{i=1}^{n}{\alpha }_{i}{p}_{i}$$

Where *a*_*i*_ is the abundance of complex *i and pi is a base (unit) vector along axis i representing the copy number of the ith protein complex*.

Each region can exhibit a different complex distribution. To analyze the differences between regions and simulations, we used principal component analysis. In the PCA outputs, the points represent the different complex abundances for each region in the dimension-reduced space allowing the largest differences between the brain regions to be visualized. Originally, each region could be assigned a vector with as many dimensons as complexes, with a value for the abundance of each complex. Our data matrix X used for PCA in our study has dimensions of 524 (regions) by 222,784 (complex types), with a rank of 524. This setup ensures that the PCA can appropriately capture the variability and structure within our data, with 524 independent components reflecting the rank of the matrix. To get more insight into the role of individual complexes in the PCA, we have determined the importance of each complex by combining two measures obtained from the standard principal component analysis: (1) the fraction of variability explained by the given principal component and (2) the contribution of the abundance of the protein complex investigated to each of the PCs. In this way it is possible to calculate how relevant and informative the original base vectors (representing complex abundances) are in the comparison of brain regions.

2 $$r\in {R}^{n},r={\sum }_{i=1}^{n}\frac{{\lambda }_{i}}{{\sum }_{j=1}^{n}\left|{u}_{ij}\right|{u}_{i}}$$

Where *r* is an *n*-dimensional vector containing the relevance of (variance explained by) each complex, *λ*_*i*_ is the relevance of the *i*-th eigenvector, *u*_*ij*_ is the *j*-th coordinate (component in the original space with a dimension for each complex) of the *i*-th eigenvector *u*_*i*_.

### Statistical comparison of complexes and regions

A pairwise T-test is a classical statistic usually chosen when only one change - here the mutation - is created in the system and the question is how the change affects the mean value. The null hypothesis is that the mean abundance of a complex (in the wild-type and mutant scenario) is the same within a certain level of significance. Thus the alternative hypothesis is that the mean abundance of a given complex formed from the protein set with the wild-type and the mutant proteins differ significantly.

The calculations were performed as implemented in the scipy package (method scipy.stats.ttestrel), where the t-score is calculated as in Eq. 3 [27]:

3 $${t}_{score}=\frac{mean\left(a-b\right)}{\sigma }$$

where *a* and *b* are 40-length arrays of the abundances of the given complex for wild-type and mutant simulations respectively and is the standard error.

The p-value is calculated from the t-score based on the alternative hypothesis type that is two-sided:

4 $$p={2cdf}_{t,39}\left(-\left|{t}_{score}\right|\right)$$

where *cdf* is the cumulative density function for the T-test with the degree of freedom 39 (for 40 repetitive simulations).

Given that the abundance of each complex is averaged separately during the simulation, the T-test shows which hypothesis we can accept for the abundance only of the given complex (*p*_*i*_).

## Results

We ran several simulations based on the Gillespie algorithm. The inputs of the simulations are protein abundances and parameters for simulating protein complex formation dynamics, along with the descriptions of the possible pairwise interactions. Protein abundance data, being highly variable as shown in Table 1 along with at PCA analysis in Figure S1. The outputs are protein complexes and their abundances for each brain region. In our simulation outputs, we have observed the formation of 222,784 different complexes.

### Identification of the most informative complex

Principal component analyses (PCA, Fig. 1b, c) show which complexes are the most informative from the point of view of distinguishing brain regions. The first two principal components for both the wild-type and mutant scenarios cover 44% and 24% of the full variance of the outputs, respectively. The first principal axis is dominated by the abundance of the AMPAR/PSD−95 (id:12) complex, whereas the second one by the PSD−95/SynGAP (id:8) complex.

The most informative complex overall – with the highest contribution considering all principal components and the variance explained by them – is AMPAR/PSD−95/SYNGAP (id:5) (Fig. 1d) with a contribution of 19%. In comparison, the importance of the average complex is very small, approximately 4.48e−06 and the median is 9.98e−08. This is because of the high number of possible complexes that can form.

### Complex composition and relevance

Common proteins in the complexes formed are PSD-95, AMPAR and SYNGAP. PSD-95 appears in all the top 10 most informative complexes, indicating its central role in complex formation. AMPAR is present in 6 out of the 10 complexes, showing its frequent participation, whereas SYNGAP is involved in 5 of the complexes, highlighting its significance. This pattern suggests a common scaffold or core structure. Despite the recurrence of certain proteins, the specific combinations and additional proteins like NMDAR and GKAP create diverse structures. Notably absent from the top 10 complexes are Shank1 and Homer1, proteins known for their ability to polymerize or heterotetramerize. Their absence suggests that Shank1 and Homer1 and the complexes that have Shank1 and Homer1 are involved in forming much larger complexes. These larger complexes tend to have lower individual occurrences, leading to reduced overall variance and relevance in this specific analysis.

### Overall complex distribution is primarily determined by protein availability

Principal component analysis of the simulation results for the wild-type and mutant scenarios show a very similar overall picture. The two PCA plots can directly be compared as the axes are the same even in the two independent PCA outputs. The data points corresponding to the wild-type and mutant cases move only minimally relative to each other (Table S6). The average distance between wild-type and mutant regions is 1.5 ± 0.8.

We note that our PCA results do not generally separate the source brain regions with the exception of the cerebellar cortex-type regions that are concentrated at a distinct region from the others (Fig. 1b, c).

Our results suggest that the overall protein complex distribution is determined by the availability of the individual proteins and the presence of a weakening mutation does not cause substantial global effects. This is in line with the system retaining its general functionality. In order to analyze the effect of the mutation in more detail, we have investigated the abundance of the individual protein complexes.

The significance of changes in the formation of the most informative complex does not seem to be closely related to the abundance of Shank1 and GKAP, the interaction of which is directly affected by the mutation (Fig. 1f). The only observable pattern is that no significant changes are found in regions with high abundance of both proteins. On the other hand, many regions with low or moderate abundance of these two proteins also do not show a significant change. This observation reinforces our conclusion that the availability of partners in the wider interaction network has a profound effect on complex formation.

### Weakening the Shank: GKAP interaction causes subtle effects in well-defined regions

To analyze the effect of the mutation introduced, we have compared the complex distributions in each region with the wild-type simulations. At the level of individual complexes, there are some exhibiting significant changes in the mutated scenario relative to the wild-type one.

For larger supercomplexes, even small numerical change in their abundance can be significant due to the very low probability of their formation. Therefore, in the case of supercomplexes, even the appearance of one complex can be considered relevant.

Importantly, the p-values of the most informative complex AMPAR/PSD−95/SynGAP drop below 0.0024 for some of the resulting data sets (Fig. 1e, Table S8). This is somewhat surprising as this complex does not contain either of the partners of the interaction affected by the mutation. Thus, it is worth investigating how the abundance of the individual partner proteins Shank1 and GKAP influence the changes observed in the formation of AMPAR/PSD−95/SynGAP complex. We have plotted the input abundance of Shank1 and GKAP vs. the p-value of the most informative complex (Fig. 1e). It is apparent that significant changes are not confined to either the high- or the low-abundance regions of the two affected proteins (Fig. 1f), suggesting that the complex interplay between the interactions of multiple proteins is behind the observed phenomenon.

As a result of the mutation, we would expect the complexes in the layer above GKAP, i.e. those containing the membrane receptors and PSD−95, to become more favored since the interaction between GKAP and Shank1 connects these complexes to the larger supercomplexes where Shank1 polymerizes. This effect is observed only in the cerebellar cortex regions and to a small extent. Some example abundances are shown in Figure S2.

The abundances change from zero to 1 for the complexes SynGAP/PSD−95/GKAP (id:9) and AMPAR/PSD−95/GKAP (id:15). However, overall complex abundances remain highly similar to the wild-type even in the regions with the smallest p-values.

Curiously, the regions with the lowest p-values all belong to the cerebellar cortex. The structure and levels of the cerebellum are significantly different compared to the cerebrum, which also means differences in the main neuron types [28]. The differences have already been demonstrated for different Shank3, Shank2 abundances in different layers in the cerebellum [29], and the different contributions of the cerebellum to ASD [30]. Naturally, we do not claim that the observed low p-values directly reflect these aspects, but it cannot escape our attention.

To get further insight into the changes of complex abundance, we have selected two regions: H376.IIIB.53M1C-S1C exhibits the lowest nonzero p-value for the most informative complex (AMPAR/PSD−95/SynGAP) (Figure S3).

In these two region, the average abundance of the complex AMPAR/PSD−95/SynGAP (id:5) changes from 306 to 301 upon the mutation. In addition, the abundance of the related complex AMPAR/PSD−95/SynGAP/GKAP increases. Thus, the decrease can be partially attributed to the fact that the complex AMPAR/PSD−95/SynGAP (id:5) associates with GKAP with a higher probability than in the case of the wild-type. The greater availability of uncomplexed GKAP can be explained by the weakened Shank1:GKAP connection.

On the other hand, for the two regions with the highest p-values (H376.XI.50HIP and H376.VIII.53MD), the abundance of the complex AMPAR/PSD−95/SynGAP does not change at all (Figure S4).

## Discussion

### Justification of our approach

Our model of only seven PSD proteins and without any specific spatial organization is definitely a highly simplified one that is far from the actual biological complexity of the postsynapse. In addition, for simplicity, we consider a situation that corresponds to a homozygous scenario, i.e. where either only wild-type or mutant Shank1 is present but not both. Last but not least, we have modeled only one well-defined effect of the mutation, ignoring possible pleiotropic effects like the alteration of the expression level of multiple proteins as observed for several Shank mutations [31]. Thus, it is not expected that the obtained protein complex distributions can be directly compared to the in vivo situations. Modeling all these aspects with acceptable accuracy would require much more data than currently available. However, we argue that our model system, focusing on a well-defined set of major PSD proteins and interactions is complex enough to capture general aspects of the behavior of elaborate protein networks with a multitude of binding interactions while remaining manageable in terms of data analysis as the number of possible protein complexes is not extremely high. On the other hand, mechanistic linking of genotypes with phenotypes, with different genotypes leading to similar phenotypes, is only possible via a combination of experimental data and modeling approaches.

The fact that the distribution of complex formation is not distinguishable in different brain regions and does not significantly change upon mutation does not necessarily indicate that the results are random. Here are some key points to consider: The cerebellum being isolated, we found distinguishable patterns, suggesting that the results are not entirely random. If the results were purely random, we would expect more significant changes across all regions, including the cerebellum. The physical binding model used in the study is deterministic, meaning that the processes it simulates are governed by specific physical laws. Only the diffusion process involves random elements, which are based on probabilistic associations and dissociations. If the dependence on inputs were truly random, it would undermine the reproducibility of the results. However, the model’s consistency with physical laws supports its reliability. The reproducibility of the results across different simulations and conditions strengthens the argument against randomness. Consistent findings across various simulations suggest that the observed patterns are driven by underlying biological processes rather than random fluctuations.

Experimental investigation of the dependence of protein complex formation on the availability and binding properties of its constituent proteins would be a challenge. In vitro reconstruction of multicomponent systems is far from routine but not unprecedented [32]. However, quantitative analysis of a number of different species formed would require elaborate methods, most likely based on mass spectrometry. In addition, the use of full-length proteins, especially membrane receptors might not be feasible, requiring the design of constructs containing only the relevant interaction sites. In an in vivo setting, immunoprecipitation or high-resolution microscopy like 3D z-stack STORM could be used at the level of individual synapses [33]. However, for a multicomponent complex the different fluorescent labeling of the constituent proteins might be an issue along with the quantification of the individual proteins and conducting the experiments in diverse brain regions.

### Weakening a specific interaction can cause limited but significant changes

Mechanistic linking mutations to the phenotypes they cause is many times a non-trivial task, especially when the mutation perturbs a highly complex protein network. This phenomenon is well known in the case of neurodevelopmental disorders, where similar observed phenotypes can be caused by a number of different mutations. For example, a recent recommendation for Phelan-McDermid syndrome puts emphasis on the underlying genetic cause as the phenotypes are largely non-specific and can generally occur in a number of neurodevelopmental diseases [11].

The specific effect of mutations can be enigmatic, especially when they affect proteins present in many different tissues. This is especially true for cell types in which even the major partners and interactions are expected to be the same. The diversity of neurons in terms of the different abundance of postsynaptic proteins offers a unique opportunity to explore the effect of specific mutations in a complex but still simplified multicomponent system having the same set of building blocks.

Our simulation-based approach, focusing on the formation of protein complexes as defined by the abundance of their constituent proteins and their interactions, reveals that the effect of a mutation weakening a specific interaction heavily depends on the availability of all interaction partners in the system. The complex interdependence of the interactions leads to a scenario where the overall changes in protein complex distributions are generally subtle, the formation of only a few complexes are significantly affected and this effect can be confined to a well-defined set of cells with specific protein abundance. While common wisdom could suggest that protein complexes containing the mutated proteins are most affected and in cells where these are abundant, our results indicate that cells with the lowest number of affected proteins can also be among the vulnerable ones, and the protein associates mostly affected are linked only indirectly to the actually weakened interaction. Our simulations suggest that the cerebellum may be an involved brain region (Figure S4), aligning with findings in the literature [28–30].

## Conclusions

Our results suggest that even for a ubiquitous occurring interaction, effects can be highly dependent on the wider context, including the availability of all components in a larger protein-protein interaction network. Our modeling, however simplified, indicate that the affected cells can not be easily predicted based purely on the abundance of the interaction partners. Modeling different scenarios with distinct protein abundances also suggest that mutations that (also) affect protein expression levels may have a more serious effect than those that moderately perturb a given interaction. In addition, the effect of redundancy in the system (e.g. Shank1-Shank3) might not only cause individual isoforms directly take over each other’s roles, but rather they reduce intermediary effects, e.g. here, the change in the frequency of the most important complex would always be moderated by the presence of a protein with a binding pattern similar to that of the mutant. We believe that with the availability of more data from single-cell studies and combining these with simulations like the one presented here, we might get closer to the understanding of cell-to-cell variability in healthy and diseased conditiions.

## Electronic supplementary material

Below is the link to the electronic supplementary material.

Supplementary Material 1

Supplementary Material 2

## Data Availability

All data used for the simulations during this study are included in this published article and its supplementary information files. To make testing our calculations and setups possible, we have set up a web interface, available at https://psdcomplexsim.cytocast.com. Other data are available from the corresponding author upon reasonable request.

## Abbreviations

PSD: Postsynaptic density
ASD: Autism spectrum disorder
PMS: Phelan-McDermid Syndrome
PCA: Principal component analysis
