# Correction to: Simulated complexes formed from a set of postsynaptic proteins suggest a localised effect of a hypomorphic Shank mutation

**DOI:** 10.1186/s12868-024-00900-0

**Published:** 2024-10-09

**Authors:** Marcell Miski, Áron Weber, Krisztina Fekete-Molnár, Bence Márk Keömley-Horváth, Attila Csikász-Nagy, Zoltán Gáspári

**Affiliations:** 1 Faculty of Information Technology and Bionics, Pázmány Péter Catholic University, Budapest, Hungary; 2 Cytocast Hungary Kft, Budapest, Hungary

**Correction to: BMC Neurosci 25, 32 (2024)**

10.1186/s12868-024-00880-1

Following publication of the original article [1], the authors identified an error in Fig. 1.

The correct figure was originally supplied, however during proofing was accidentally replaced by the authors with an incorrect version of the figure.

The incorrect Figure 1 is given hereafter:

**Fig. 1:**
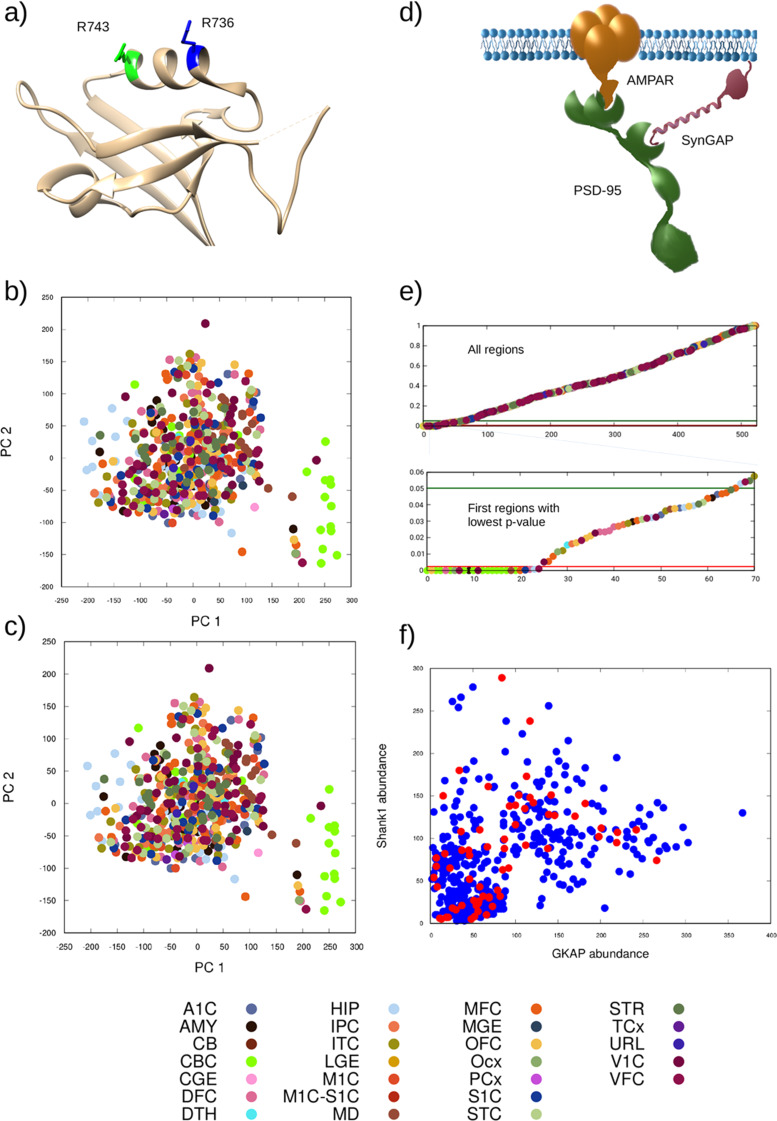
(**a**) Position of the mutation selected (R743H, green) and a similar one reported in ASD (R736Q, blue) on the ribbon representation of the Shank1 PZ domain (PDB ID 6YWZ). Both arginines are located on the α2 helix flanking the ligand binding groove. Principal component analysis of the obtained protein complex distributions for (**b**) the wild-type and (**c**) the mutant scenarios investigated. Different colors denote different brain regions according to the key at the bottom. (**d**) Schematic depiction of the most informative complex according to the PCA (AMPAR/PSD-95/SynGAP). (**e**) P-values describing the change upon the mutation relative to the wild-type, the value for the most informative complex is shown in increasing order from left to right, colored by the region type (key at the bottom) The green line denotes the 0.05 significance limit while the red line the limit of 0.0024 obtained using the Benjamini-Hochberg correction. (**f**) Abundance of Shank1 and GKAP, the two proteins in the interaction affected by the mutation, in the input data sets. Red circles indicate data sets where the abundance of the most informative complex changed significantly in the output using the Benjamini-Hochberg correction

The correct Figure 1 is given hereafter:

**Fig. 1:**
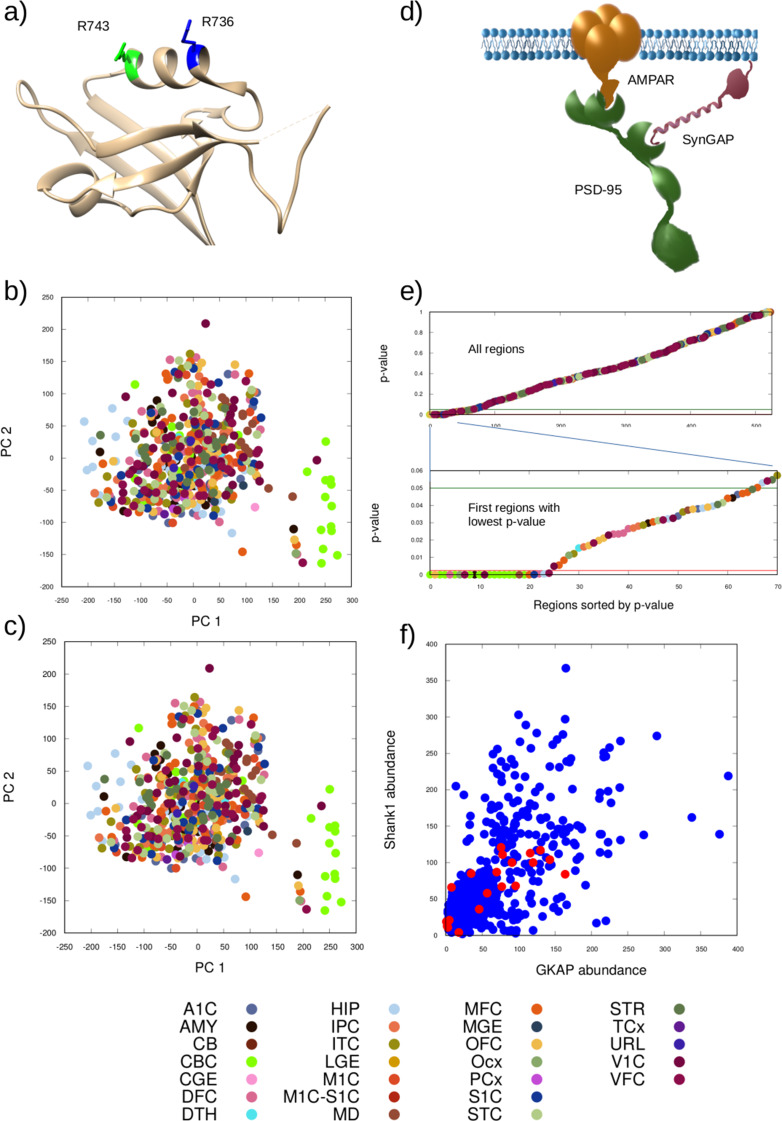
(**a**) Position of the mutation selected (R743H, green) and a similar one reported in ASD (R736Q, blue) on the ribbon representation of the Shank1 PZ domain (PDB ID 6YWZ). Both arginines are located on the α2 helix flanking the ligand binding groove. Principal component analysis of the obtained protein complex distributions for (**b**) the wild-type and (**c**) the mutant scenarios investigated. Different colors denote different brain regions according to the key at the bottom. (**d**) Schematic depiction of the most informative complex according to the PCA (AMPAR/PSD-95/SynGAP). (**e**) P-values describing the change upon the mutation relative to the wild-type, the value for the most informative complex is shown in increasing order from left to right, colored by the region type (key at the bottom) The green line denotes the 0.05 significance limit while the red line the limit of 0.0024 obtained using the Benjamini-Hochberg correction. (**f**) Abundance of Shank1 and GKAP, the two proteins in the interaction affected by the mutation, in the input data sets. Red circles indicate data sets where the abundance of the most informative complex changed significantly in the output using the Benjamini-Hochberg correction

The revised Fig. 1 has been updated in this correct article and the original article [1] has been corrected.
