# Supplementary material for: Simulated complexes formed from a set of postsynaptic proteins suggest a localised effect of a hypomorphic Shank mutation
Source: BMC Neurosci. 2024 Jul 6;25:32. doi: 10.1186/s12868-024-00880-1 (PMC11227168; doi:10.1186/s12868-024-00880-1)
Supplement: Supplementary file 2 — Supplementary Material 2 [file 12868_2024_880_MOESM2_ESM.pdf]

Supplementary material for

Simulated complexes formed from a set of postsynaptic proteins suggest a localised effect of a hypomorphic Shank mutation

Marcell Miski[1], Áron Weber[1,2], Krisztina Fekete-Molnár[1], Bence Márk Keömley-Horváth[1,2], Attila Csikász-Nagy[1,2,*], Zoltán Gáspári[1,*]

[1]Faculty of Information Technology and Bionics, Pázmány Péter Catholic University,Budapest, Hungary
[2]Cytocast Hungary Kft.,Budapest,Hungary

Supplementary methods

Simulations of complex formation

Our overview of the Gillespie algorithm follows our previously published one (Miski et al. 2022).

The detailed description of our algorithm is provided in our previous publication, but for the reader's convenience, it has also been included in the supplementary material of this paper.

Cytocast implements a version of the Gillespie algorithm, a Monte Carlo-based method to simulate every reaction in order to have a non-deterministic approach of abundance change of molecules or in our case complexes.

Main steps of the Gillespie-algorithm (Erban 2007):

1. Set the initial conditions as the number of starting molecules (here protein abundance) $n_{i,0}$ and possible reaction numbers q
2. Generating two random variables between 0 and 1. (r_1, r_2)
3. Compute the propensity functions of each reaction (here binding, note if a binding occurs there is one less protein).

$$\alpha_i(t) = (n_{i,t-1} - 1) k_i$$

4. Compute the propensity function for the whole system:

$$\alpha_0 = \sum_{j=1}^{q} \alpha_j(t)$$

5. Compute the time when the next chemical reaction takes place as $t+\tau$ where:

$$\tau = \frac{1}{\alpha_0} \ln\left(\frac{1}{r_1}\right)$$

6. Calculate which reaction occurs then adjust the numbers of the proteins (one less protein from the inputs and one more for the output (complex)). The ith reaction occurs if the conditions are true:

$$\frac{1}{\alpha_0} \sum_{j=0}^{i-1} \alpha_j(t) \leq r_2 \leq \frac{1}{\alpha_0} \sum_{j=0}^{i} \alpha_j(t)$$

The equilibrium dissociation constant (K_D) is the ratio of unbinding (k_{off}) to binding rate (k_{on}).

Cytocast is a Gillespie-based stochastic modeling software of agent-based protein-binding in a virtual cell where the proteins are point-like. Its aim is to provide a quantitative prediction on the abundance of protein complexes formed under certain initial conditions. These conditions are the simulation time, the compartments' size and shape, protein abundances, diffusion rates, protein functions, and bindings. Cytocast is based on the publicly available SiCompre software (Rizzetto 2018).

As it is unrealistic to have binding and unbinding rates for all possible reactions in the system, we set these rates uniformly to 1 a.u. (binding = 1 a.u., unbinding = 1 a.u.). Previous works have shown that this simplification can lead to biologically relevant results (Rizzetto 2015, Rizzetto 2018).

We have tested 10, 40, 50, and 100 repetitions. The standard deviations were saturated around 1 relatively quickly, meaning that 40 repetitions proved to be sufficient.

There is no theoretical limit for the number of molecular species. The practical limit is the diversity of molecules; there were no issues even with several hundreds of thousands of different kinds of particles. Usually, the overabundance is the problem, as over a couple of ten million requires more powerful hardware with higher computational power.

Data sets

Data set preparation was performed as described in our previous work (Miski et al. 2022).

Briefly, data taken from Roy et al. 2017 were processed by an in-house Python script. The RNA-Seq RPKM (reads per kilobase per million) values were used to estimate the number of protein molecules within a single synapse:

- The median RNA-Seq value of the PSD-95 mRNA were calculated as 66.82 rpkm.
- The average number of PSD-95 molecules was estimated as 300 (Cheng 2006)
- The emerging ratio of 300/66.82 was used to scale the mRNA data for for all proteins and regions, assuming linear relationship based on the PhD Thesis of Madeline Farley (Farley 2015)

Assumption of binding rates by NeEMO

Specifically, the NeEMO method uses sequence and structure information which is transformed into a residue interaction network. The residue interaction network is further processed for example by the Dijkstra algorithm and generates input data for a trained neural network that predicts the $\Delta\Delta G$ for a given mutation.

The $\Delta\Delta G$ refers to the entire protein, not domain-specific. As a result, the disadvantage is that the changes in all bonds of a given protein are treated equally and only estimated from the degree of change in stability.

The change rate of the unfolding rate ($K=\frac{[denatured]}{[natured]}$) can be derived from $\Delta\Delta G$. The change rate tells how the unfolding rate changes by the mutation according to the predicted $\Delta\Delta G$ values assuming that the ΔG is in equilibrium.

$$\Delta\Delta G = -RTln(K_{WT}) + RTln(K_{MT})$$

$$\text{change rate} = \frac{K_{MT}}{K_{WT}} = e^{\frac{\Delta\Delta G}{RT}}$$

The probability of complex formation is inversely proportional to the change of unfolding rate in our model thus the change_rate shows how much the unbinding_rate changes.

Supplementary Figures

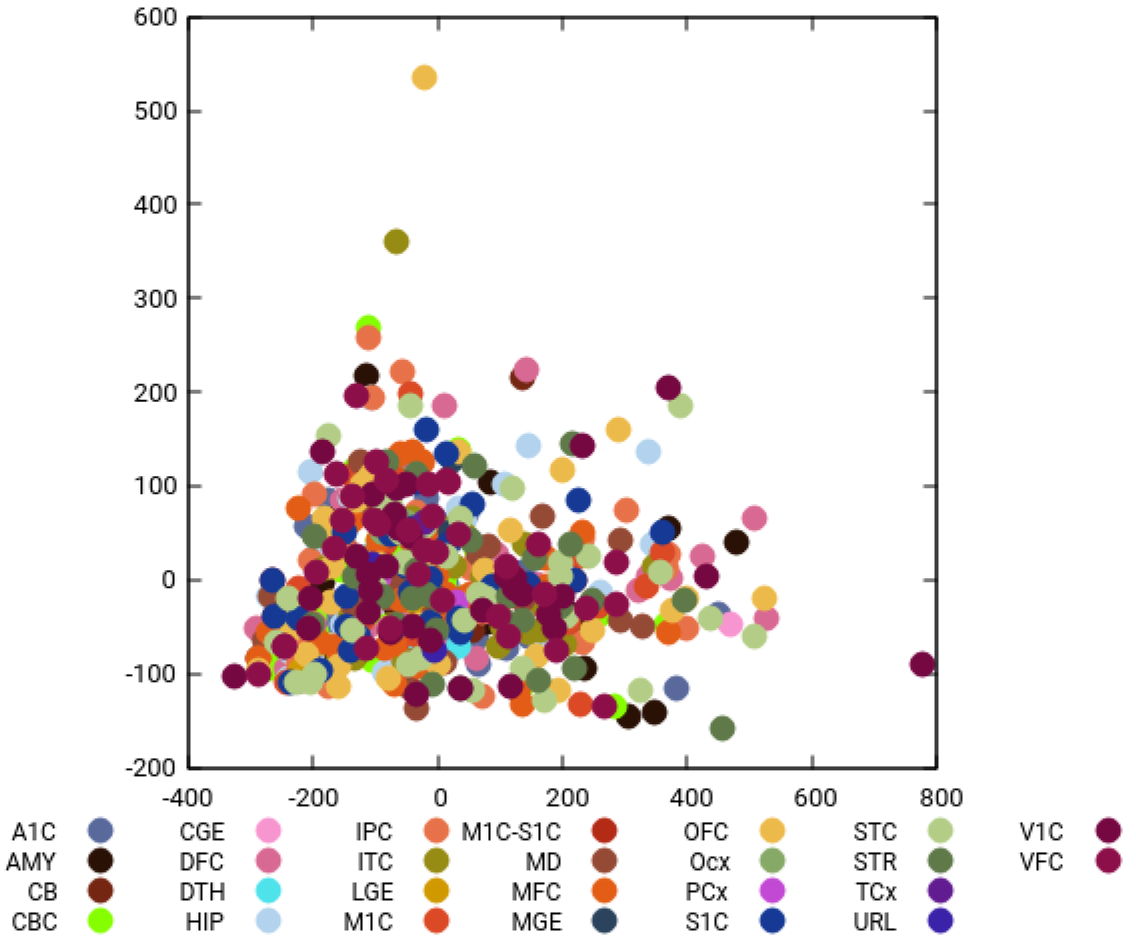

Figure S1. Principal component analysis of the 524 brain regions investigated based on the input protein abundance data (7 proteins). The abbreviations denote the types of the regions. There is no clear pattern by region type for the protein abundances.

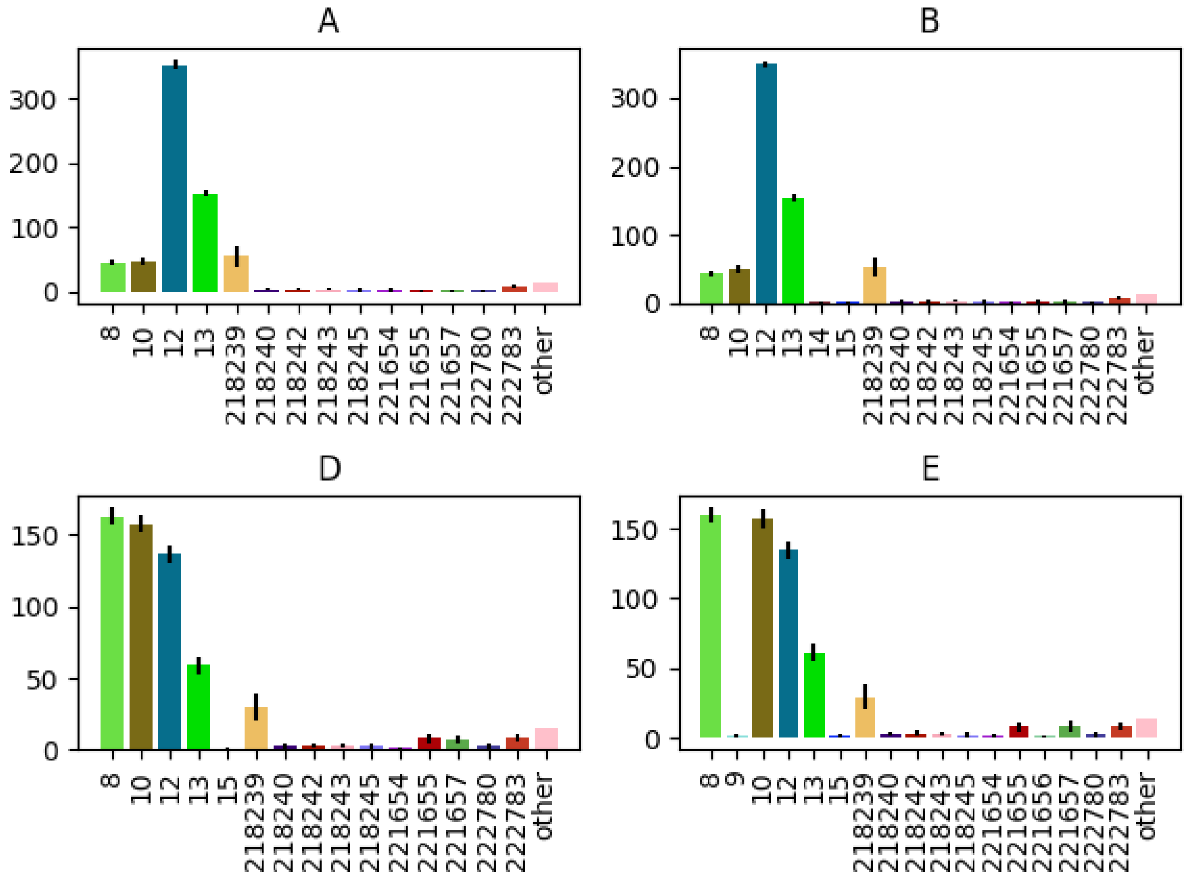

Figure S2. Complex distribution in selected cerebellar regions.
Distribution of protein complexes in the H376.XI.50_CBC and H376.XI.52_CBC regions for the wild-type and mutant scenarios.
A) H376.XI.50_CBC wild-type B) H376.XI.50_CBC mutant
C) H376.XI.52_CBC wild-type D) H376.XI.52_CBC mutant.
The complexes depicted are:
0:NMDAR/PSD-95;
1:NMDAR/PSD-95/SYNGAP;
4:PSD-95/AMPAR;
5:PSD-95/AMPAR/SYNGAP;
6:PSD-95/AMPAR/SYNGAP/GKAP;
8:PSD-95/SYNGAP;
10:PSD-95/GKAP;218239:GKAP/Shank1;
218240;218242 and 218243: variations of GKAP/Shank1/SHank1;
221654:GKAP/Shank1/Homer1;
222783:Homer1-tetramer

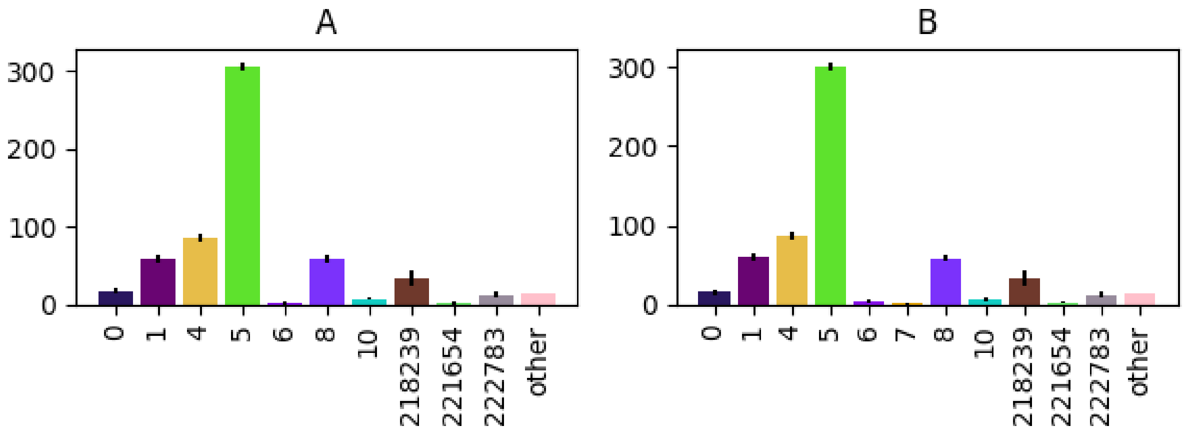

Figure S3. Distribution of protein complexes in the H376.IIIB.53_M1C-S1C region for the wild-type and mutant scenarios
A) wild-type B) mutant
The complexes depicted:
0:NMDAR/PSD-95;
1:NMDAR/PSD-95/SYNGAP;
4:PSD-95/AMPAR;
5:PSD-95/AMPAR/SYNGAP;
6:PSD-95/AMPAR/SYNGAP/GKAP;
8:PSD-95/SYNGAP; 10:PSD-95/GKAP;
218239:GKAP/Shank1;
221654:GKAP/Shank1/Homer1;
222783:Homer1-tetramer

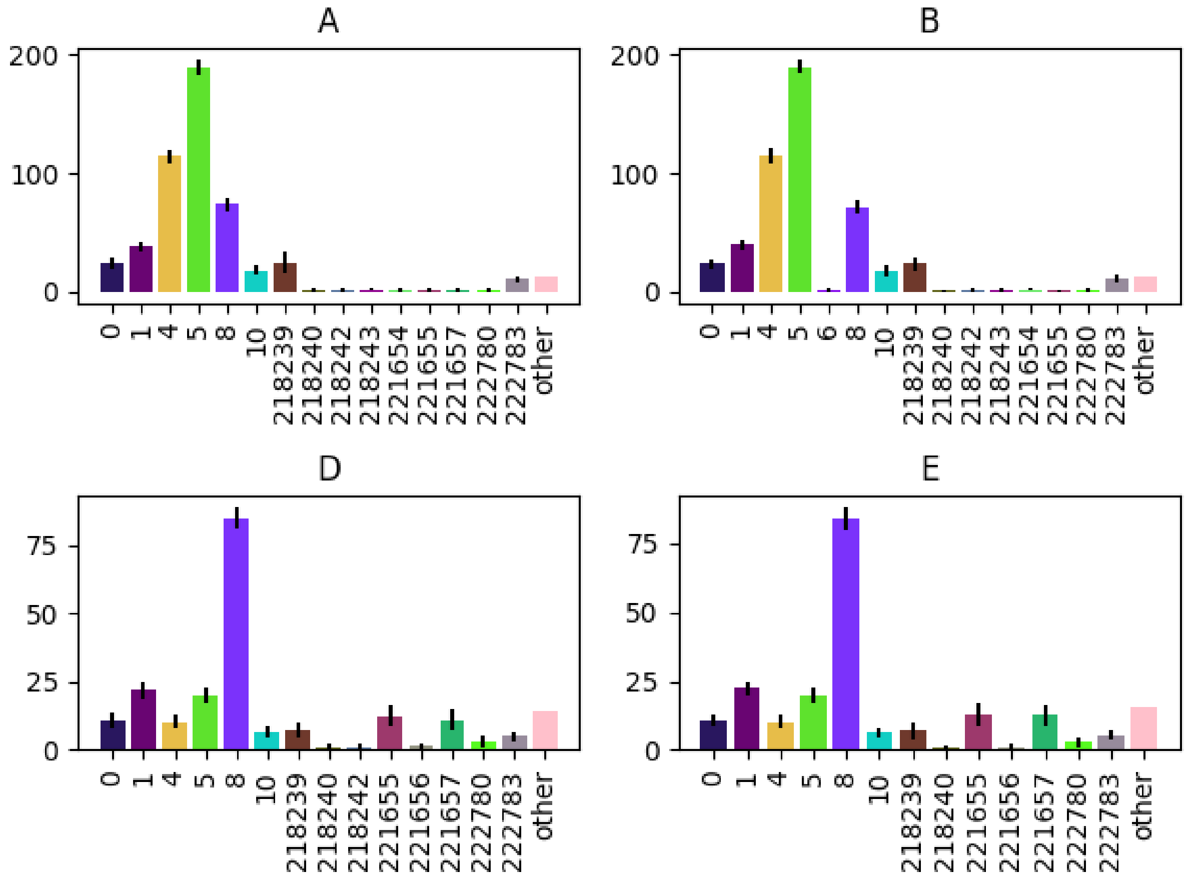

Figure S4. Distribution of protein complexes in the H376.XI.50_HIP and H376.VIII.53_MD regions for the wild-type and mutant scenarios. A) H376.XI.50_HIP wild-type B) H376.XI.50_HIP mutant C) H376.VIII.53_MD wild-type D) H376.VIII.53_MD mutant.
The complexes depicted:
0:NMDAR/PSD-95;
1:NMDAR/PSD-95/SYNGAP;
4:PSD-95/AMPAR;
5:PSD-95/AMPAR/SYNGAP;
6:PSD-95/AMPAR/SYNGAP/GKAP;
8:PSD-95/SYNGAP;
10:PSD-95/GKAP;218239:GKAP/Shank1;
218240;218242 and 218243: variations of GKAP/Shank1/SHank1;
221654:GKAP/Shank1/Homer1;
222783:Homer1-tetramer

Supplementary references

Cheng D, Hoogenraad CC, Rush J, Ramm E, Schlager MA, Duong DM, et al. Relative and absolute quantification of postsynaptic density proteome isolated from rat forebrain and cerebellum. Molecular & Cellular Proteomics. 2006; 5:1158–1170. https://doi.org/10.1074/mcp.D500009-MCP200

Erban R, Chapman SJ, Philip, Maini K. A practical guide to stochastic simulations of reaction-diffusion processes. 2017. http://arxiv.org/abs/0704.1908

Farley M.: Structure and composition of postsynaptic densities. The University of Texas MD Anderson Cancer Center UTHealth Graduate School of Biomedical Sciences Dissertations and Theses. 2015. https://digitalcommons.library.tmc.edu/utgsbs_dissertations/603/

Miski M, Keömley-Horváth BM, Rákóczi Megyeriné D, Csikász-Nagy A, Gáspári Z. Diversity of synaptic protein complexes as a function of the abundance of their constituent proteins: A modeling approach. PLoS Comp Biol 2022. 18:e1009758. https://doi.org/10.1371/journal.pcbi.1009758

Rizzetto S, Priami C, Csikász-Nagy A. Qualitative and quantitative protein complex prediction through proteome-wide simulations. PLoS Comp Biol. 2015;11:e1004424. https://doi.org/10.1371/journal.pcbi.1004424.

Rizzetto S, Csikász-Nagy A. Toward large-scale computational prediction of protein Complexes. In: Methods in Molecular Biology. Springer New York; 2018. p. 271–295. https://doi.org/10.1007/978-1-4939-8618-7_13.
